# A Survey on Graph Neural Networks for Microservice-Based Cloud Applications

**DOI:** 10.3390/s22239492

**Published:** 2022-12-05

**Authors:** Hoa Xuan Nguyen, Shaoshu Zhu, Mingming Liu

**Affiliations:** 1 Insight SFI Research Centre for Data Analytics, Dublin City University, Dublin 9, D09 DX63 Dublin, Ireland; 2 School of Electronic Engineering, Dublin City University, Dublin 9, D09 DX63 Dublin, Ireland

**Keywords:** anomaly detection, graph neural networks, microservices, resource scheduling, software decomposition

## Abstract

Graph neural networks (GNNs) have achieved great success in many research areas ranging from traffic to computer vision. With increased interest in cloud-native applications, GNNs are increasingly being investigated to address various challenges in microservice architecture from prototype design to large-scale service deployment. To appreciate the big picture of this emerging trend, we provide a comprehensive review of recent studies leveraging GNNs for microservice-based applications. To begin, we identify the key areas in which GNNs are applied, and then we review in detail how GNNs can be designed to address the challenges in specific areas found in the literature. Finally, we outline potential research directions where GNN-based solutions can be further applied. Our research shows the popularity of leveraging convolutional graph neural networks (ConGNNs) for microservice-based applications in the current design of cloud systems and the emerging area of adopting spatio-temporal graph neural networks (STGNNs) and dynamic graph neural networks (DGNNs) for more advanced studies.

## 1. Introduction

Currently, the Internet of Things (IoT) has become an essential element in all aspects of human life. With different connected sensors in place, data collected from these devices can be transmitted to an IoT edge-cloud-empowered analytics platform to provide various applications and services to benefit citizens in cities [1,2,3,4]. Deep learning, with its ability to learn from the massive amount of data from the cloud through neural networks, can perform better analysis for various data-driven problems, which cannot be easily achieved using conventional statistical approaches [5,6].

However, with such an enormous volume of IoT data to be collected and processed, it is less beneficial in practice to deploy monolithic applications in a cloud platform particularly considering their scalability and performance. In contrast, a microservice architecture, which consists of a collection of small, independent, and scalable services, has become the new paradigm for the design and development of cloud-native applications. The key idea of the architecture is to allow a group of microservices working together to achieve common goals through efficient communication protocols and mechanisms facilitated in cloud networks.

**Figure 1:**
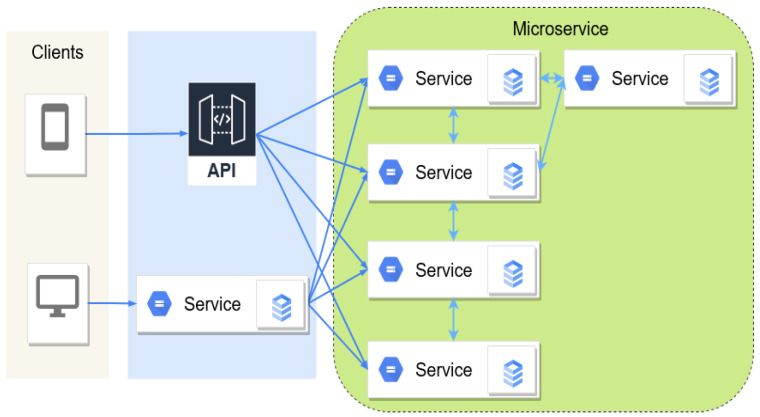
A typical microservice architecture.

At present, microservice architectures have been widely deployed by many cloud computing giants, such as Microsoft, Google, and Amazon [7]. This architecture, as shown in Figure 1, allows software developers to factorize a monolith application into multiple small components, providing flexible configurations and resource management for cloud-native applications. Each microservice can be independently designed, developed, and deployed, which further enables data-driven and machine-learning-based functions, such as predictive maintenance and elastic scaling, to be supported in the cloud systems [8,9].

The workload of an application in the cloud is dynamic, which dramatically increases the complexity of the design of autoscaling algorithms. Machine-learning-based methods have demonstrated their efficacy in scaling [10,11] with a faster response [12] compared to reactive rule-based strategies [13]. Machine-learning models can learn and interact with the environment and automatically adapt to dynamic workloads [14]. This natural property of machine-learning-based methods is suitable for the scalability decision of cloud applications. Moreover, some authors combined a set of validated machine-learning methods to further improve the accuracy and efficiency of the algorithms in different scenarios [15,16,17].

In [15], a combination of the convolutional neural network (CNN) and boosted trees (BT) methods was proposed by Zhang et al. The model analyses the dependencies of the microservices, the tail latency quality of service (QoS), and long-term QoS violations. This approach abstracts the complex microservice connection solely using collected data (the resource usage history, latency history, and resource allocation).

Yan et al. [16] proposed a combination of the bidirectional long short-term memory (Bi-LSTM) load prediction model and active SARSA reinforcement learning model to obtain a more accurate workload prediction and to improve the resource utilization while ensuring service level agreement (the response latency of the service) and the total resource limit. The resource-allocation algorithm utilized both reactive and active methods; however, the dependencies between microservices were not considered in the decision-making process.

Moreover, Park et al. [17] showed how the dependence of the microservice chain is important for accurate monitoring of the cascade effect and can be used in an autoscaler to make decisions satisfying the latency service-level objective (SLO). The authors collected the front-end workload and distributed the workload of each microservice as inputs for training the graph neural network model using the gradient-descent method, where the aim was to predict the minimal CPU quota of the corresponding microservice.

Graph neural networks and their variants have been applied to address graph-based learning challenges in various domains, including but not limited to, physical system modelling [18,19], chemical reaction prediction [20], biological disease classification [21], traffic state prediction [22,23,24,25], text classification [26], machine translation [27], and object detection [28,29].

To illustrate a few, the authors in [23] devised an attention-based temporal graph convolutional network to predict anomalous lane-changing behaviours of drivers in highway scenarios. In addition, the authors in [27] proposed an encoder–decoder architecture coupled with a gated graph neural network for graph-to-sequence learning problems arising in natural-language processing.

Most recently, GNNs have also been utilised for microservice-based applications due to their good properties linked to graph data structures. A “microservice graph” can be used to represent the dependencies of microservices in an application. In a calling graph, the GNN models can capture the back-pressure and cascading effect as GNNs learn the dependencies of the graph via messages passing between nodes. GNNs can be adopted to build sophisticated analytic frameworks for modelling and predicting the metrics of applications in cloud systems, e.g., workload characteristics, system resource utilization, and network throughput.

In addition, GNNs can also capture both the spatial and temporal dependency of a dynamic microservice graph where the node attributes change over time through transfer learning. These attributes along with the naturally interconnected graph of the services in the microservice architecture make GNN an ideal method to handle the irregularity and dynamic resource needs of microservices. Currently, there are ongoing research efforts in applying GNNs to microservice-based applications on anomaly analysis [30,31,32,33,34], resource scheduling [17,35,36], and refactoring monolith applications [37,38].

Various GNN algorithms have been utilised in microservice-based applications to further improve the prediction accuracy and computational efficiency. However, we found a lack of studies summarizing the key GNN applications for microservice architecture. Therefore, this paper focuses on the current literature in which GNNs and their variants were applied to analyse complex issues in microservices. This leads to the specification of the potential research topics and extensive research questions for applied GNN-based microservice studies. To summarize, our contributions are:

1. We investigate the current development and application of GNNs for microservice tasks.
2. We identify the key research areas where GNNs can be applied to microservices.
3. We summarize the main technical challenges and research gaps in relation to the applications of GNNs for microservices.
4. To the best of the authors’ knowledge, this is the first review paper that attempts to highlight this new and advanced field of research where GNNs can be leveraged for microservice-based applications.

The rest of this paper is organized as follows. Section 2 reviews the related survey papers in the literature. Section 3 introduces our review technique. Section 4 briefly recaps the background of graph neural networks and their design variants. Section 5, Section 6 and Section 7 review a collection of microservice-based applications using graph neural networks. Section 8 discusses the current challenges and indicates potential research directions in the future based on the findings of this review work. Finally, Section 9 concludes the paper.

## 2. Related Survey Papers

This section reviews the current survey papers on GNNs to provide context for our work. There are several survey articles on GNNs investigating the development of GNNs, reviewing their corresponding methods, and presenting practical applications in various disciplines [29,39,40,41,42] as shown in Table 1. These surveys highlighted the ongoing interest in applying GNNs for solving graph-based problems; however, there are still research challenges that remain. For instance, these aforementioned surveys did not provide a thorough analysis of each problem. These surveys also did not elaborate on the proposed GNN algorithms involved therein. In addition, in the survey papers, there is a lack of comparison between the proposed approaches despite their similar research questions.

Moreover, the existing surveys either focused on the design of microservice-based applications or cloud-system taxonomy [43,44] without considering applications of GNNs to cope with existing challenges in the microservice discipline. Others investigated the application of machine-learning algorithms in cloud structures [14,45] with little focus on GNN algorithms. For instance, Duc et al. [45] depicted a taxonomic classification of reliable resource provisioning with details of machine-learning-based methods. Similarly, Zhong et al. [14] reviewed state-of-the-art machine-learning-based techniques for container orchestration for behaviour modelling and prediction as well as resource provisioning. However, none of the aforementioned studies details the application of GNNs and their variants for the management and resource provisioning of complex microservice-based applications.

Therefore, our key objective in this paper is to comprehensively review the state-of-the-art GNN applications where the GNN algorithms can be applied to microservices in various ways, including abstracting the service dependencies, forecasting the workload changes, and supporting refactoring of the monolith applications. We hence aim to establish an extension of the existing surveys to review recent advances in GNNs for addressing challenges in microservice-based applications. The settings of GNN models in such applications are studied in detail in our review. We also outline the datasets and tools associated with each application.

## 3. Review Methodology

We conducted the literature survey process in five phases: planning, researching the literature, reporting and interpreting the results, highlighting the challenges, and making recommendations as per the practice in [46]. The following parts of this section detail the key research questions to be addressed and our process of the literature search.

### 3.1. Research Questions

The survey sought answers to the following questions in relation to the application of GNNs in microservice architecture:

Q1: How many papers have been published regarding GNNs for microservice-based applications?

Q2: How can GNNs be modelled and designed to cope with microservice applications?

Q3: What are the main purposes of using GNNs for microservice applications?

Q4: What are the main advantages and disadvantages of using GNNs for microservice applications, particularly in comparison to other machine-learning-based techniques?

Q5: What are the key challenges and research gaps in further advancing GNNs for microservice applications?

### 3.2. Literature Search

We collected research papers that used GNNs to address microservice problems from publications in peer-reviewed research. We screened the relevant papers through an extensive review of the literature on problems of microservice structure to be solved, the design of GNN models and problem setups. We used various online databases that index computer science and technology research—namely, Taylor and Francis, PubMed, ACM Digital Library, Springer, MDPI, IEEE, Science Direct, Nature, PLOS one, and Elsevier.

The search keywords were: graph neural networks, microservice, microservice anomaly detection, microservice resource scheduling, and monolithic decomposition. We also used synonyms to complete some of the keywords, i.e., we used a cloud-based application in place of a microservice. Only papers that were written in English were considered in our search. Additionally, we only used articles using GNNs for microservice applications. For all papers that shared the same group of authors or the same title, we only used the most recent papers. Table 2 details the inclusion and exclusion criteria in our process of screening relevant studies. Through the screening and eliminating process, we found 10 papers (five for anomaly detection, three for resource scheduling, and two for software decomposition) for review as tabulated in Table 3.

The results show that no existing survey paper in the literature focused on the applications of GNNs for microservice architecture. The results also indicate that GNNs have been applied for addressing anomaly detection, resource scheduling, and software decomposition-related challenges in microservice-based applications. The existing studies focused largely on the application of GNNs for building graphs connecting individual services and establishing a correlation among services. All selected papers for review are relevant in our context, which highlights the applicability of GNNs in dealing with microservice problems in different aspects.

## 4. Graph Neural Networks

In this section, we review the basics of GNNs. A GNN is a type of deep neural network that is suitable for analysing graph-structured data. A graph is typically represented by a set of *V* vertices (nodes) and a set of *E* edges (links), G=(V,E), where a vertex $v_{i}\in V$ and a directed edge $e_{ij}=\left(v_{i},v_{j}\right)\in E$ between $v_{i}$ and $v_{j}$ is represented by an arrow as i→j, forming an ordered pair of nodes $(v_{i},v_{j})\in V\times V$ in a directed graph. Therefore, the number of nodes is denoted by n=|V|, and the number of edges is m=|E|.

A vertex *u* is a neighbour of the vertex *v* in the graph G=(V,E) if there is an edge (v,u)∈E,∀u∈V. We define N(v) as the set of neighbours for the node *v* in the graph *G*. The connection between nodes can be represented by an adjacency matrix $A^{n\times n}$ with $A_{ij}=1$ if $e_{ij}\in E$ or $A_{ij}=0$ otherwise. We define the node attribute matrix $X\in R^{n\times d}$ with $x_{v}\in R^{d}$, where *d* denotes the dimension of $x_{v}$, representing the feature vector of a node *v*. In addition, a graph can be associated with an edge attribute matrix $X^{e}\in R^{m\times c}$ with $x_{v,u}^{e}\in R^{c}$ representing the feature vector of an edge (v,u).

Undirected edges are assumed to have equal weights from both connected nodes, while the directed graphs have all edges directed from one node to another. A spatial-temporal graph is a special case as the node attributes change with time, i.e., $G^{\left(t\right)}=\left(V,E,X^{\left(t\right)},X^{e(t)}\right)$, where the node attributes and edge attributes can be time-varying.

The GNN architecture can be sorted into four main categories: recurrent graph neural networks (RecGNNs), convolutional graph neural networks (ConvGNNs), graph autoencoders (GAEs), and spatio-temporal graph neural networks (STGNNs) [39]. Except for RecGNNs, the defined architectures differentiate from each other in how graph convolution layers and functionality layers are stacked [39]. Regarding RecGNNs, which aim to learn node representations, they assume that a node in a graph exchanges information with its neighbours until a stable state is reached. The idea of message passing is a crucial concept resulting from RecGNNs.

**Figure 2:**
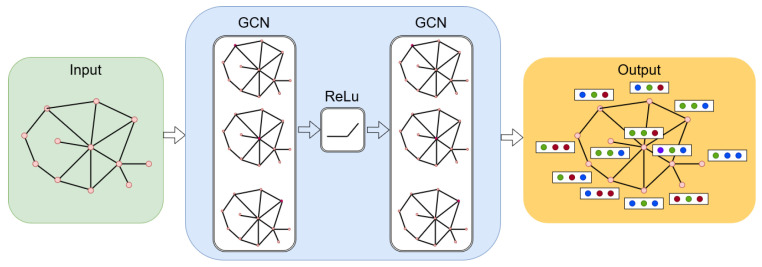
Illustration of graph convolutional neural networks.

ConvGNNs extract high-level node representation by stacking multiple graph convolution layers, which can be used for either node representation or graph representation problems. A node *v* representation is generated by aggregating its own features $x_{v}$ and its neighbouring features $x_{u}$, where u∈N(v). An example model based on ConvGNNs is shown in Figure 2. GAEs are unsupervised learning frameworks that are used to learn network embedding and graph generation. This method abstracts the characteristics of nodes or graphs into a latent vector space. The encoded vector is then used to reconstruct graph data using the activation function. Hence, the models learn from the minimization of the discrepancy between the real graph properties and the reconstructed properties, i.e., an adjacency matrix for network embedding.

Dynamic graph neural networks (DGNNs) refer to the use of GNNs for dynamic graphs where the nodes and edges may vary over time [47]. Dynamic graphs can be sorted into discrete-time dynamic graphs (DTDGs) and continuous-time dynamic graphs (CTDGs). A DTDG is represented by a sequence of graph snapshots $\left[G^{\left(1\right)},G^{\left(2\right)},...,G^{\left(T\right)}\right]$ where each graph at time *t* is defined by $G^{\left(t\right)}=\left(V^{\left(t\right)},A^{\left(t\right)},X^{\left(t\right)}\right)$. A CTDG is defined by its initial state $G^{\left(t_{0}\right)}$ and its temporal observations (O) describing the evolution of $G^{\left(t_{0}\right)}$ in a time-dependent manner [48].

STGNNs, which are a special case of DTDGs, target the hidden properties of the spatial-temporal graphs such that both spatial dependency and temporal dependency can be captured from the data in graphs simultaneously. This characteristic is particularly useful to address practical challenges, such as traffic-flow prediction, as data generated in a traffic network can be easily modelled using a spatial-temporal graph. In this context, for instance, a graph convolutional network (GCN) layer can be applied to learn the spatial dependency between nodes, e.g., sensors on the streets, and a long short-term memory (LSTM) layer can be applied afterward to capture the temporal dependency [49].

A graph instance *G* with node features ($x_{v}$) and edge features ($x_{\left(u,v\right)}^{e}$) are the input of the GNN model. The hidden state of the nodes is represented by $h_{v}$. The hidden state of the edges is denoted by $h_{\left(v,u\right)}$. The initial state of the node is assumed as $h_{v}=x_{v}$. A node or an edge representation in a hidden state is recurrently updated by the exchange of information of the central node with its neighbours by

(1) $$h_{\left(v,u\right)}=f_{edge}\left(h_{v},h_{u},x_{\left(v,u\right)}\right)$$

(2) $$h_{v}^{\prime }=f_{node}\left(h_{v},\sum_{u\in N(v)}h_{\left(u,v\right)},x_{v}\right),$$

where $f_{node}$ and $f_{edge}$ are recurrent functions [41]. By updating $h_{v}\leftarrow h_{v}^{\prime }$, GNN models can provide various graph-level outputs: node-level outputs, edge-level outputs, and graph-level outputs [39,41].

GNNs can extract high-level node-level outputs, such as representations for node regression or node classification tasks using a multi-perceptron or a softmax layer as the output layer. At the node level, the classification tasks aim to sort the nodes of the graph into classes, while the prediction tasks are to forecast the continuous values of nodes in the graph. Node clustering groups similar nodes into the same group. For the edge-level outputs relating to edge classification and link-prediction tasks, GNNs can be utilized to determine the connection strength of an edge or to predict a link between two nodes.

The edge-level tasks also focus on the classification of edges. At the graph level, the outputs usually relate to the graph classification task, graph regression, and graph matching. GNN layers are incorporated with pooling layers and/or readout layers to abstract a representation on the graph level. In addition, the performance of GNNs can be significantly improved by integrating the recent advances in attention mechanisms. We refer the readers to [29,39,40,41] for a detailed and rigorous review of taxonomy, variants, and computing perspectives for GNN methods.

GNNs have many applications across multiple areas of research involving classification, regression, and clustering problems; for example, node classification, node embedding [18], graph classification, graph generation [50,51,52], node prediction, graph prediction tasks [53], and node clustering tasks [54]. In this paper, we mainly focus on the study of GNNs for microservice-based applications, including anomaly detection [30,31,32,33,34,55], resource scheduling [17,35,36], and refactoring monolith applications [37,38,56]. More specifically, we detail node and edge representations of the GNN approaches in Table 4. The table also summarises microservice-based applications and public datasets used in the literature to facilitate comparative studies in the microservice domain.

**Figure 3:**
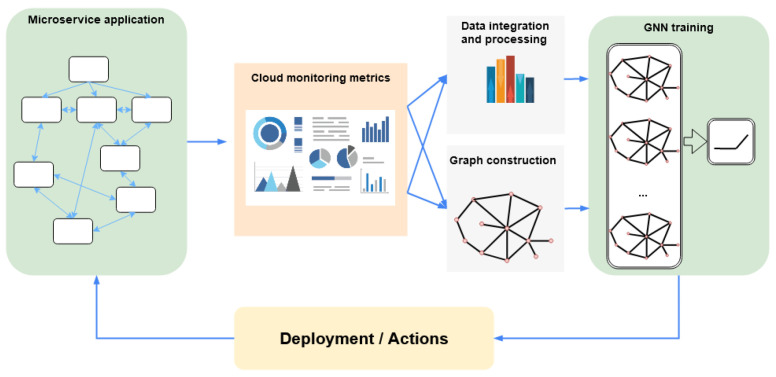
High-level flow diagram of GNN applications for microservices.

To conclude this section, Figure 3 illustrates a general pipeline for applying GNNs to microservice-based applications. In a nutshell, various performance metrics are first collected from the microservices in their deployment environment. The gathered data are then used to construct graphs for GNN-based learning tasks. The trained GNN model can be integrated with the microservice architecture to make decisions as per the specific objective of the task. In the following sections, we shall discuss GNNs for microservice-based applications in the three identified categories in detail.

## 5. GNNs in Anomaly Detection

Anomaly detection is a critical mechanism for identifying abnormal behaviours in system states or application performance. Table 5 describes the reviewed approaches regarding anomaly detection. ConvGNN and RecGNN models were applied in several studies [30,31] to generate the node representation by aggregating the central node features and its neighbouring features. In addition, STGNNs were utilised in [32,33,34] to consider both the temporal and spatial dependencies of dynamic microservice-based applications and characters of the nodes, edges, and graphs.

**Figure 4:**
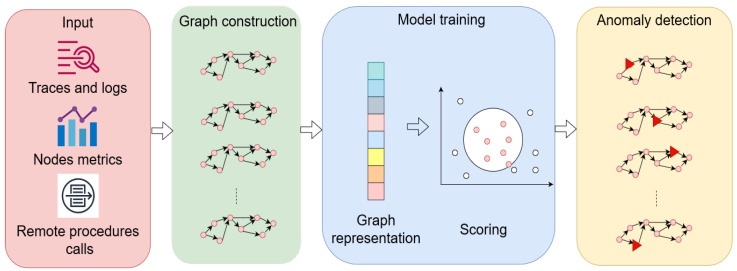
General procedure for an anomaly-detection algorithm using GNNs for microservice architecture [30,31,32,33,34].

The anomaly detection problem is modelled as a graph problem in which the microservice graphs with nodes and edges are regularised differently (i.e., a node is a call between RPC in [34], or a node is a log event in [31]). A high-level workflow abstraction from the related studies is shown in Figure 4.

Somashekar et al. [30] classified anomalous microservice using a two-stage approach. The first stage uses a four-layer GNN model to classify potential anomalous traces. The predicted potential traces are then used as the input for the second prediction model. The second model is comprised of three GCN layers to detect bottleneck microservice in these potential anomalous traces. The only feature is the service time, which correlates well with the bottleneck occurrences. The proposed approach was validated with dynamic workload profiles and microservice test bed applications [57,58,71], and the numerical results were compared with [71] for three datasets of various complexity.

However, the splitting structure of the proposed algorithm is prone to hierarchical errors, since the results of the second model are dependent on the classified outputs from the first model. This was shown in the predicted results provided by the classification models with a better prediction if the number of anomalous traces was large in the dataset, i.e., the number of traces in the dataset with bottleneck microservice was larger than the number of traces without bottlenecks. This also might result from the semi-supervised framework of the bottleneck prediction model. On the other hand, the prediction models were proposed with empirical numbers of GCN layers, which might not be applicable for other datasets or problems. In addition, the model is static so it is trained using offline data and cannot adapt to online data for continuous updates on the model.

Gated GNNs (GGNNs) were utilized to learn the trace representation—namely, a trace event graph (TEG), constructed using both the log events and span events of a trace and their relationships [31]. The GGNN algorithm computes the vector latent representation of each node (event) using node attributes (event vector) and, consequently, the graph representation of the TEG. From that, the unsupervised model calculates an anomaly score of the TEG using the graph representation to classify whether the trace is anomalous. The proposed method was tested with data collected from Train Ticket [58], a microservice-based application, and its fault cases. This study illustrates the advantage of GGNNs in abstracting the node and graph representation for a complex microservice structure. The proposed method relies on the construction of a unified graph representing traces and logs rather than the improvement of the GNN method itself. On the other hand, the applicability of this approach is dependent on the preprocessing task where the traces and logs are used to generate an overall graph, which might not be possible for a real-life microservice structure.

He et al. [32] proposed an unsupervised anomaly-detection algorithm combining GNN and LSTM for predicting anomalies in cloud systems. The proposed model modifies LSTM cells with additional GNN gates to replace fully connected layers in LSTM to construct a GraphLSTM cell. This design extracts a temporal dependence among contaminated data (with both normal data and abnormal data) using the LSTM structure, while added GNN gates utilise predefined topological representations containing features of each node. The GNN gates provide an edge topology characteristic array to incorporate the spatial properties of the service architecture. By sliding the monitoring window through the collected metrics, the temporal and spatial dependence are considered in the prediction results.

Data for training and testing are collected from the microservice-based application, i.e., Hipster Shop [61]. There are clear effects of utilizing GNN to model the spatial dependence among components in a cloud system as illustrated by comparing to free GNN-based models. Although the combination of two advanced machine-learning methods provided promising results, it is worth noting that the topological information was represented by a static edge set array, which, in turn, constrained the applicability of the proposed method to static applications. A possible improvement is a real-time topology, which could change with a dynamic workload and requests.

Another two-step approach was proposed by Chen et al. [33]. First, the authors proposed an identifier to determine the RPC chain pattern using density-based clustering techniques. A static RPC graph was generated by a set of related RPCs at a given time step. The second task was the construction of a prediction model for each critical RPC chain pattern given a time series of graphs. Each node of the static graph represents an RPC with the node attribute being RPC traffic, i.e., the number of calls in a fixed time period.

The edges are set with each edge representing an RPC dependency. The model, therefore, predicts anomalies based on the observations of the RPC traffic from the previous time history. To generate the prediction for attributes of each node, the spatial-temporal GCN model is used. The author utilised the diffusion convolution recurrent neural network (DCRNN) to model RPC chain graphs to learn the hidden patterns from the spatial-temporal graph and to make predictions.

The predictions are made on the selected RPC graph, which differentiates this method from DeepTraLog [31] whose graph is built for a unified graph of the overall microservice structure. This makes this approach compact since it only considers a static RPC chain at a time instance, instead of the whole RPC chain patterns. On the other hand, the RPC chain pattern must be assumed to be static during a sufficiently long time period to construct the RPC graph. Therefore, the predicted results for each RPC depend on the assumed RPC graph instance and, thus, ignore the dynamic inter-cluster dependencies.

Jacob and colleagues [34] also proposed a prediction model using a DCRNN, a spatial-based ConvGNN, for cyber security attacks in a microservice-based applications. This study was motivated by [33]; however, only one learned model was used to learn the entire microservice-based application, while the former required an updated model periodically. The main difference between this method and the former is that the topology of the application is represented as a weighted directed graph, where a node is a call from one RPC to another RPC. To represent the directed graph, the adjacency matrix is predefined prior to forecasting the traffic. The method leverages the DCRNN model to capture the temporal dependencies of the microservice traffic. The proposed model then can predict the number of times an RPC is called; however, this removes the adaptivity of the [33] due to its predefined adjacency matrix.

## 6. GNNs in Resource Scheduling

In this section, we discuss various resource provisioning techniques proposed to dynamically adjust the system resource in response to the workload and cloud environments. Table 6 shows various GNN algorithms to solve the scheduling issues.

**Figure 5:**
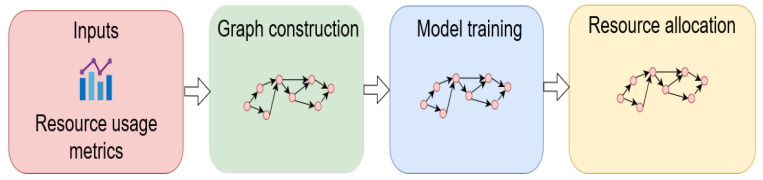
General procedure for resource-allocation algorithms using GNNs for microservice-based cloud applications.

For resource scheduling, various GNN algorithms are utilised for different purposes, i.e., learning the characteristics of different microservices for forecasting resource consumption. The GNN-based proactive algorithms yield much more accurate results compared to the reactive rule-based scheduling methods in these cases—that is, system resources in the cloud systems can be scheduled dynamically and allocated to the workload prior to changes. Figure 5 illustrates the general steps for the development of resource-allocation algorithms using GNNs for microservices in the literature.

Park et al. [17] proposed an autoscaler that allocates resources to every microservice according to the change in the front-end workload. In the microservice graph, a node represents a microservice, and the workload and allocated CPU resources are the node features. The GNN model comprises a message-passing neural network (MPNN) for graph node embedding and fully connected layers for latency prediction. By utilising GNN to elicit node embedding, a vector that implies information from all neighbouring nodes, the model updates the node states by recurrently exchanging neighbouring microservice information.

The two consecutive fully connected layers predict the latency of each microservice and make resource allocation for every microservice. The end-to-end latency of an application was computed based on the longest microservice chain of the application. Only the in-line microservices in the longest chain were considered. Therefore, the cross-line microservices (i.e., the microservices out of the longest chain) were neglected. A two-stage predictive algorithm—namely, Graph-PHPA—was recently proposed by Nguyen et al. [35] in 2022.

To improve the resource allocation quality of the horizontal autoscaler, the proposed approach aimed to investigate the dependence of the microservices chain effect with a strong focus on resource allocation from the perspective of cloud service providers. More specifically, the approach investigated the graphical dependence of microservices on resource consumption with respect to their workloads.

The approach focused on addressing the resource SLO challenge, which is a fundamental difference compared to [17]. The author integrated the predicted workloads using LSTM and the resource consumption model based on GCN to proactively best estimate the desired amount of resources for each microservice. The scaling algorithm, which considers the real-time workloads and the available resources in the cluster, could make an accurate scheduling decision on optimal vCPU resources to be provisioned for microservices in the cluster.

A bipartite graph neural network (Bi-GNN) with a bidirectional message-passing mechanism was utilized by Hou et al. [36] to infer the time-varying characteristics of microservices. The GNN model was applied to extract the node representation in terms of the dynamic graph of the microservice. The graph is represented by two disjoint sets of vertices (two microservice sets) and a set of directed edges from one vertex set to another. The microservice feature is their resource requirement. Each application is considered a bi-particle graph that represents the basic characteristics, such as unevenly distributed resource demands and interactions of the component microservices of an application.

The authors also proposed a bi-partite attention mechanism that iteratively updates the inferred features to reflect the uneven effects on each other between the API and function microservices. Based on a set of inferred features that specify the required resources the microservice requires, the optimal resource managing strategies allocate the resource to effectively adapt to resource requirements from all possible allocation strategies. This method’s advantage is that the resource classifier was trained offline. However, as a bi-particle graph can change dynamically with time due to real-life workload, the classification model must be retrained to adapt to the workload profile.

## 7. GNNs in Software Decomposition

**Figure 6:**
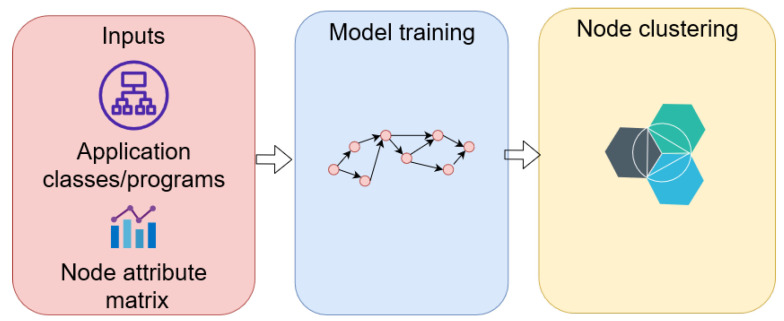
General procedure for application refactoring algorithms using GNNs for microservice architecture.

As depicted in Table 7, GNNs are also utilized for software decomposition, a complementary mechanism for refactoring monolith applications to microservice-based applications in cloud environments. By characterizing monolith programs and their relationships in the application as a graph and using GCN layers to derive node representation, microservice decomposition becomes a clustering task leveraging graph representation learning. Figure 6 depicts a high-level description for application refactoring algorithms with GNNs in the reviewed studies.

To refactor a monolith structure to a microservice structure, Desai et al. [37] designed a software-decomposition method considering the class in the application as a node. There is an edge between two nodes if a class directly calls a method of another class. Assisted with a modified loss function in the fundamental GNN model—namely, CO-GCN—their approach produced a more accurately unified node representation, outlier node detection, and node clustering for refactoring four examples of monolith applications. To be specific, a two-layered graph convolution encoder was used to obtain a representation of each node.

Then, a GCN-based decoder mapped the node embeddings to the input feature space. Finally, the nodes in the graph were clustered into a predefined number of clusters by minimizing a proposed loss function. In such a way, the proposed approach became an optimization problem by minimizing the total loss function comprised of three components: loss with respect to the structural outlier, loss of the reconstruction of node attributes, and loss to cluster the nodes in the graph. While the authors proposed a novel framework that generated candidate microservices, the algorithm still requires human interference with a predefined number of clusters, where each cluster performs a well-defined functionality.

To further extend the Desai et al. [37] approach, Mathai et al. [38] further included application resources for recommending a microservice architecture. The refactoring monolith application problem was treated as a heterogeneous graph model with function nodes and resource nodes. The complete network included four GCN layers to find a network embedding for each node: the first two layers as encoders (compressing the feature space) and the next two as decoders. The design was to exchange features between nodes and edges and update the vector representation for both.

Therefore, an attribute matrix was generated using aggregated features from all related function nodes into the resource nodes to form the resource attribute matrix, and this enabled the representation of application data resources and programs of the monolith application. The final set of node attributes included an attribute matrix for the program node, a resource attribute matrix, and an edge attribute matrix. Then, community-aware heterogeneous graph neural network (CHGNN) was chosen to find the optimal complete microservice network with a desired number of microservices through the GNN training process. An encountered problem is that the empirical number of predefined clusters was still not dealt with in this study.

## 8. Research Directions and Challenges

**Figure 7:**
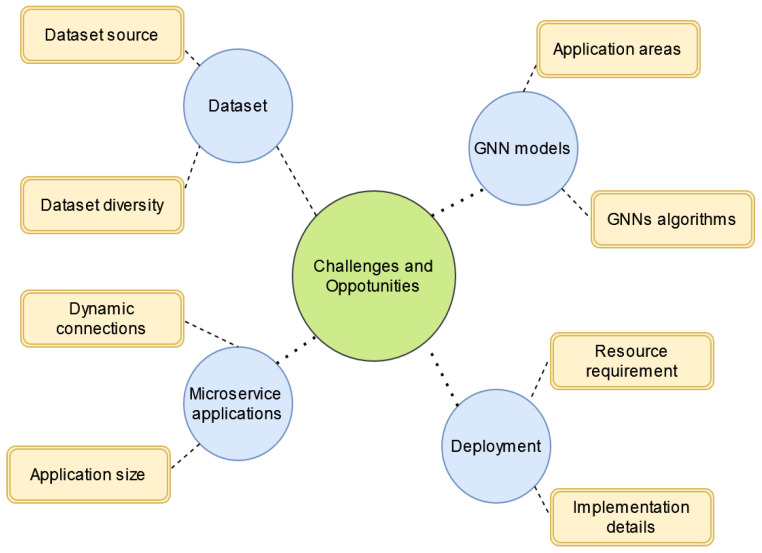
The challenges and opportunities for GNNs for microservice architecture.

Although GNNs have proven their advantages in learning microservice-based applications, there are still challenges due to the complexity of microservice graphs and the limitations of GNN models. Thus, in this section, we suggest some future directions to indicate potential research gaps in GNN models for microservice applications. Our key findings are summarised and illustrated in Figure 7. More specifically, we identify several open questions that might inform future research regarding GNN models, the deployment of GNN models, microservice applications, and public microservice datasets.

As the internal structure of microservice applications is complex and dynamic with continuously growing demands for more accurate predicted results, lower response times, and better computational costs, this requires robust and efficient GNN models to be trained and deployed in real-world scenarios.

First, the success of an application lies in the proposed GNN models. As can be seen from Table 5, Table 6 and Table 7, most of the current works have utilized ConvGNNs for microservice tasks; however, there are a limited number of works employing RecGNNs, STGNNs, and GAEs despite having achieved promising results in other domains. It is worth considering applying these GNN variants to tackle the challenges arising in microservice-based applications from different perspectives. Furthermore, attention-based mechanisms have been widely applied in other application domains with strong links to GNN-empowered designs as they have demonstrated promising results in performance improvement; see papers, such as [23,49,72,73,74,75]. However, limited efforts have been made regarding integrating attention mechanisms with GNNs for microservice-based applications. Our review demonstrated that [55] is perhaps the only work in this endeavour.

Another design aspect is the tuning of the model hyperparameters. For instance, as indicated in [76], an increasing number of hidden layers might degrade the performance of a ConvGNN dramatically. Therefore, how to determine the optimal hyperparameters to deal with a learning task whilst considering both performance metrics and computational resource consumption is an important area for GNNs applied to microservices. Last, but not least, the selection of a directed or an undirected graph is an important decision variable particularly when the data to be analysed is collected from an application in a less observable environment, i.e., the logical structure of the application is not known a priori. Thus, an effective modelling tool is required to understand the structure of the graph before a detailed analysis can be implemented.

Finally, according to the studies that we reviewed in this work, the microservice graph is always assumed to be static. However, real-time interconnection among microservices is often ignored leading to less-accurate modelling of the graph for real-world scenarios. For this purpose, a dynamic graph may be useful, and currently more research efforts are required to dive into this challenging area.

Secondly, microservice architecture has attracted significant attention due to its flexibility and adaptability. The application structures are also flexible and complex, which, in a generalized graph, can contain different types of definitions for nodes/edges as well as various forms of inputs for nodes/edges. For example, a node may represent a service deployed on a cluster and the call between two services is the edge in [32]. Although certain works have been found that deal with the challenges in heterogeneous graphs, more novel GNN architectures and solutions are required to deal with increasingly complex scenarios, such as temporal graphs and multi-dimensional input metrics.

Finally, microservice-based applications are dynamic in nature. Nodes and edges may exist or disappear depending on the functionality of calls; therefore, node/edge inputs may vary with time. This requires an instant update on the graph structure to capture the actual connectivity and attributes of nodes. Although STGNNs may be used to address this challenge (for instance, by considering the inputs to the graph during every fixed time window), the dynamic characteristics remains to be modelled, which may otherwise lead to performance degradation in certain learning tasks. Furthermore, future works are required for modelling the continuous-time dynamic graph of microservice networks; for instance, the application of CTDGs, which offer the ability to model finer-grain temporal graph changes in continuous time [47].

## 9. Conclusions

In this paper, we reviewed works in the literature where GNNs were applied for microservice-based applications. Depending on the architecture of the GNNs, each variant had particular advantages for use in specific applications to further improve the modelling and prediction results. We summarized the GNN models and their key benefits linked to microservice-based applications in three key areas, which were identified as anomaly detection, resource scheduling, and software decomposition.

In these areas, GNNs have shown their ability to perform well in various tasks, such as node classification, node prediction, and graph construction. Finally, we made suggestions for future research directions, where we remark that there has been little focus in applying STGNNs and DGNNs for microservice applications, which may help to further improve such systems in a variety of practical scenarios.

## Figures and Tables

**Table 1:** Summary of recent related surveys.

Ref.	Microservice Architecture	GNNs Classification	GNNs Applications	Benchmark Dataset	Brief Description
[39]		x	x	x	Wu and colleagues [39] applied GNNs to model graph-based data from public datasets and categorised GNNs into four subgroups, including recurrent graph neural networks (RecGNNs), convolutional graph neural networks (ConvGNNs), graph autoencoders (GAEs), and spatio-temporal graph neural networks (STGNNs).
[29]		x	x	x	Zhou et al. [29] generalised the design pipeline for GNN applications and classified GNN variants using computational modules, graph types, and training settings. The applications of GNNs were grouped into two scenarios: structural and non-structural scenarios. Popular platforms and open-source codes of referred papers are also provided.
[40]		x	x		Sato [40] particularly focused on the mathematical formulations of GNNs to explain their capability and applicability more than on practical applications.
[41]		x	x	x	Waikhom and Patgiri [41] categorised GNNs using the perspective of supervision and provided a valuable table of the benchmark dataset for various GNN applications.
[42]		x	x	x	The authors [42] focused on GNNs from the computation aspect and provided a comprehensive overview of software and hardware support for GNNs.
This paper	x	x	x	x	The aforementioned studies did not give comprehensive reviews on the application of GNNs to the microservices structure. We aim to provide a comprehensive survey of existing research papers that utilise GNNs variants to resolve microservice-based applications.

**Table 2:** The literature inclusion and exclusion criteria.

Inclusion Criteria	Exclusion Criteria
GNN application for microservice architecture	GNNs for other applications
Full-text	Uncompleted studies
Published at any time	Research not published in English
Published in the aforementioned databases	Duplicated studies, the same group of authors
Published in workshops, symposiums, conferences, books, and journals	Non peer-reviewed publications

**Table 3:** Related papers.

Title	Author	Type	Year	Venue
B-MEG: Bottlenecked-Microservices Extraction Using Graph Neural Networks	Somashekar et al. [30]	Conference	2022	Companion of the 2022 ACM/SPEC International Conference on Performance Engineering (ICPE 2022 Companion)
DeepTraLog: Trace-Log Combined Microservice Anomaly Detection through Graph-based Deep Learning	Zhang et al. [31]	Conference	2022	44th International Conference on Software Engineering (ICSE 2022)
A Spatiotemporal Deep Learning Approach for Unsupervised Anomaly Detection in Cloud Systems	He et al. [32]	Conference	2020	IEEE Transaction on Neural Networks and Learning Systems
Informer: Irregular Traffic Detection for Containerized Microservices RPC in the Real World	Chen et al. [33]	Journal	2022	High-Confidence Computing
Anomalous Distributed Traffic: Detecting Cyber Security Attacks Amongst Microservices Using Graph Convolutional Networks	Jacob et al. [34]	Journal	2022	Computers & Security
Graph-PHPA: Graph-based Proactive Horizontal Pod Autoscaling for Microservices using LSTM-GNN	Nguyen et al. [35]	Conference	2022	IEEE International Conference on Cloud Networking 2022
GRAF: A Graph Neural Network based Proactive Resource Allocation Framework for SLO-Oriented Microservices	Park et al. [17]	Conference	2021	The 17th International Conference on emerging Networking EXperiments and Technologies (CoNEXT 2021)
AlphaR: Learning-Powered Resource Management for Irregular, Dynamic Microservice Graph	Hou et al. [36]	Conference	2021	2021 IEEE International Parallel & Distributed Processing Symposium
Graph Neural Network to Dilute Outliers for Refactoring Monolith Application	Desai et al. [37]	Conference	2022	The Thirty-Fifth AAAI Conference on Artificial Intelligence (AAAI-2021)
Monolith to Microservices: Representing Application Software through Heterogeneous Graph Neural Network	Mathai et al. [38]	Conference	2022	International Joint Conference on Artificial Intelligence

**Table 4:** Graph structure and dataset.

Ref.	Year	Nodes	Edges	Task	Graph	Toolkit	Applications and Dataset
[30]	2022	Microservice	Calls	Node-level	Unweighted	NA	DeathStarBench [57]; TrainTicket [58]; FIRM public dataset [59]
[31]	2022	Span events	Calls	Graph-level	Weighted	PyTorch Geometric [60]	TrainTicket [58]
[32]	2020	Services	Calls	Graph-level	Weighted	PyTorch Geometric [60]	Hipster shop [61]; HiBench [62]
[33]	2022	Remote procedure calls (RPCs)	RPC dependency	Graph-level	Weighted	TensorFlow [63]	Authors in-house data
[34]	2022	RPCs	RPC dependency	Graph-level	Weighted	TensorFlow [63]	DeathStarBench [57]
[35]	2022	Microservice	Calls	Node-level	Weighted	PyTorch Geometric [60]	Bookinfo [64]
[17]	2021	Microservice	Calls	Node-level	Unweighted	PyTorch Geometric [60]	Online Boutique [65], Social Network [57]
[36]	2021	APIs/ function services	The dependencies of API microservices and function microservices	Graph-level	Weighted	Python programs	TrainTicket [58]; DeathStarBench [57]; Alibaba trace dataset 2017 [66]
[37]	2022	Classes of function	Calls between two classes	Graph-level	Weighted	Python programs	Daytrader [67], Plantsbywebsphere [68], Acme-Air [69], Diet App [70]
[38]	2022	Classes of function	Calls between two classes	Graph-level	Unweighted	PyTorch Geometric [60]	Daytrader [67], Plantsbywebsphere [68], Acme-Air [69]

**Table 5:** Summary of anomaly-detection approaches.

Ref.	Methods	Mechanism	Category	Objectives	Advantages	Limitations
[30]	DGCNN	Classification	ConvGNNs	Classifying potential anomalous traces. Detect anomaly microservices in such traces.	Improved prediction accuracy. Better performance with a fairly imbalanced dataset without any feature engineering.	Static model. Only used one data feature: service time. Hierarchical error.
[31]	Gated-GNN-SVDD	Classification	RecGNNs	Building trace event graph (TEG) consists of the log events and span events of a trace and their relationships. Microservice trace anomaly detection.	Unified application graph using traces and log events. Improved detection accuracy.	Limited microservice structure. Insufficient evaluation of real-life application-level anomalies.
[32]	GCN-GNN-LSTM	Classification	GAEs	Anomaly detection and prediction on application metrics. Including system topological information to organize metrics.	Capturing the temporal dependence of the resource metrics. Including the topological information to represent a system state.	Static model. Anomalies were injected randomly during the experiments.
[33]	DCRNN	Classification	STGNNs	Predicting future traffic. Detection of unusual changes in RPC.	Various robustness analysis. Construction of spatial-temporal graph convolution network DCRNN models for all possible critical RPC chain patterns.	Computational cost due to the generation of models.
[34]	DCRNN	Classification	STGNNs	Prediction of future traffic activity. Detecting cyber security attacks.	A spatial-temporal graph convolution network DCRNN model for all critical RPC chain patterns. Improved detection accuracy and time-consumption.	Static graph model.

**Table 6:** Summary of scheduling approaches.

Ref.	Methods	Mechanism	Category	Objectives	Advantages	Limitations
[35]	LSTM-GCN	Regression	ConvGNNs	A two-stage proactive resource-allocation framework. Resource utilization and cost saving	Numerical simulations with practically dynamic workload profile Optimize container deployment time	Only considered the vCPU usage. Without comparison with other algorithms.
[17]	MPNN	Regression	STGNNs	Derivation of a proactive resource-allocation framework. Predicting expected end-to-end tail latency for the front-end workloads and possible CPU resource allocations.	Accounting for cascading effect. Allocating required resources while satisfying latency SLO.	Implicit computational cost. Simple workload profile.
[36]	Bi-GNN	Classification	ConvGNNs	Abtracting microservice-based application using bipartite graph. Resource utilization optimization.	Various resource features. Manage resources adapting the load variation.	Insufficient analysis of computational costs and time complexity.

**Table 7:** Summary of software decomposition approaches.

Ref.	Methods	Mechanism	Category	Objectives	Advantages	Limitations
[37]	CO-GCN	Clustering	ConvGNNs	Refactoring a monolith application into microservices. Utilizing GNN to group nodes into clusters.	Representing applications using graph structure. Using the node representation for clustering.	Neglecting application data resources. Implicit computational cost.
[38]	CHGNN	Clustering	RecGNNs	Refactoring a monolith application into microservices. Using a heterogeneous graph to represent software structure with data entities.	Resource contention consideration.	Lack of consideration on load balancing.

## Data Availability

Not applicable.
